# Role of Sirtuins in the Pathogenesis of Rheumatoid Arthritis

**DOI:** 10.3390/ijms24021532

**Published:** 2023-01-12

**Authors:** Agata Poniewierska-Baran, Oliwia Bochniak, Paulina Warias, Andrzej Pawlik

**Affiliations:** 1 Institute of Biology, University of Szczecin, Felczaka 3c, 71-412 Szczecin, Poland; 2 Department of Physiology, Pomeranian Medical University in Szczecin, Powstancow Wielkopolskich 72, 70-111 Szczecin, Poland

**Keywords:** sirtuins, SIRT, rheumatoid arthritis, RA, pathogenesis, musculoskeletal system

## Abstract

Rheumatoid arthritis (RA) is an autoimmune and inflammatory disease leading to joint destruction. The causes of RA are not fully known. Most likely, the development of the disease depends on the coexistence of many factors, such as hereditary factors, immune system defects, gender, infectious agents, nicotine, and stress. Various epigenetic changes have been identified and correlated with the aggressive phenotype of RA, including the involvement of sirtuins, which are enzymes found in all living organisms. Their high content in the human body can slow down the aging processes, reduce cell death, counteract the appearance of inflammation, and regulate metabolic processes. Sirtuins can participate in several steps of RA pathogenesis. This narrative review presents, collects, and discusses the role of all sirtuins (1–7) in the pathogenesis of rheumatoid arthritis.

## 1. Introduction

Rheumatoid arthritis (RA) is a chronic inflammatory disease that affects the joints and various organs. Its most characteristic symptoms are pain, stiffness, and swelling in the joints of the hands and feet, but other joints may also be inflamed [1]. If untreated, the disease most often leads to joint damage and severe disability, as well as damage to many organs and premature death. Early application of effective treatment slows the progression of the disease, prevents complications, and allows normal functioning. Various epigenetic changes have been identified in recent years and correlated with the activation of an aggressive phenotype in RA, including DNA methylation, histone modifications, miRNA, X-chromosome inactivation, bromodomains, as well as sirtuins (SIRTs) [2]. Due to the fact that SIRTs enzyme proteins control cellular metabolism and the aging process, the role of the seven sirtuins in many age-related diseases began to be studied, such as cardiovascular diseases, diabetes type II, cancer, as well as RA. Sirtuins are important proteins that can affect the body’s immune cell responses, including T and B lymphocytes and neutrophils, but also macrophages and chondrocytes, which are directly involved in the development of inflammation in RA. To date, most is known about the role of SIRT1 in RA, while the functions of six other SIRTs are currently being investigated.

## 2. Rheumatoid Arthritis

Rheumatoid arthritis has a destructive effect on the lining of the synovial joints, leading to progressive disability and premature death, as well as the associated socio-economic burden [3]. RA is a systemic disease that affects not only the joints but also other organs. In addition to relatively common benign lesions, such as rheumatoid nodules or sicca syndrome, there may be (although very rarely) severe complications leading to premature death, e.g., stroke or heart attack [4]. Rheumatoid arthritis is an autoimmune and inflammatory disease, affecting approximately 1% of the population, that typically affects the same areas on both sides of the body. Initially, they are the small joints of the hands and feet, but as the disease progresses, many other joints become involved. Inflammation of one large joint (e.g., the knee or shoulder joint) is an unusual but possible onset of the disease. At the onset of the disease, general flu-like symptoms such as feeling weak, low-grade fever, muscle pain, loss of appetite, and weight loss are common. They may precede or accompany the joint symptoms.

There is a fundamental difference between diagnosing RA and classifying RA. Rheumatoid arthritis diagnosis is based on pattern recognition, so clinical knowledge plays an important role in the diagnostic process of individual patients [4]. Early treatment of rheumatoid arthritis is essential for the successful control of the disease. It is associated with less severe joint damage and better functioning in patients. RA is characterized by constant, inexorable progression of lesions with periods of exacerbation and remission. Unfortunately, each exacerbation causes further damage to the joints. The chronic and crippling nature of the disease, as well as the limited effectiveness and side effects of therapeutic treatment, leads to various disorders in the somatic sphere, in mental processes, and in social activity. For this reason, the treatment of patients should be based on a multidisciplinary approach that attempts to deal with the various problems encountered by these patients, both in terms of functional and psychosocial interactions.

In the affected joints, synovial cells, fibroblast-like synoviocytes, and macrophage-like synovial cells are able to release enzymes that degrade cartilage and underlying bone tissue, as well as cytokines that increase the infiltration of immune cells [5]. An effective method of inhibiting the progression of rheumatoid arthritis is to activate the process of apoptosis and simultaneously inhibit the proliferation, invasion, and migration of RA synoviocytes fibroblasts (FLs), which would inhibit synovitis and alleviate joint deformity. Long-term immune dysregulation and the inflammatory response associated with the course of RA may lead to an increased risk of cancer development [6,7,8]. Scientific reports have described a higher risk of lung cancer [9] and non-Hodgkin’s lymphoma [10] in patients with rheumatoid arthritis. SIRTs are a promising candidate molecule in many autoimmune diseases, as well as in cancer. In 2012, Nakayama et al. [11] showed that resveratrol can inhibit synovial membrane hyperplasia, a critical factor in rheumatoid arthritis, and induce apoptosis of MH7A cells in a SIRT-dependent manner. This was the first paper to initiate further research and analysis on the action of SIRTs and their role in the pathogenesis of RA.

## 3. Sirtuins Family

Seven mammalian SIRTs (SIRT1-7) are a family of NAD+-dependent deacetylases (class III histone deacetylases) signaling proteins involved in metabolic regulation and biological processes such as cell survival, apoptosis, proliferation, cellular senescence, stress response, genome stability, and metabolism [12]. They have been confirmed for a variety of phylogenetic species. SIRTs are found in different areas of the cell, i.e., SIRT1 and SIRT2 are located in the nucleus and cytoplasm (with the possibility of circulation between), SIRT3-5 in mitochondria, SIRT6 in the nucleus, and SIRT7 in the nucleolus. The participation of SIRTs has been described in endocrine signaling, glucose homeostasis, aging, and longevity, as well as circadian control and mitochondrial biogenesis [13].

In the inflammatory microenvironment, energy metabolism is profoundly altered, and importantly, many energy metabolism sensors have been confirmed to have immunoregulatory properties [13]. It has been shown that SIRT activity has the potential for treating many age-relating diseases, such as type II diabetes, cardiovascular disease, cancer, and rheumatoid arthritis [14,15,16]. SIRT targets can affect immune cells and immune responses to modulate the progression of chronic autoimmune and/or inflammatory diseases. Many clinical observations and metabolomic analyses have revealed metabolic complications in RA patients. Therefore, the SIRT family may be important regulators of clinically relevant cellular processes that play an important role in rheumatoid arthritis [12]. There is a lot of incomplete information about the impact of SIRT on the pathogenesis of RA and residual information as a factor associated with the development of RA. According to the literature, SIRTs can control the erosive destruction of articular cartilage and bone by controlling the differentiation, maturation, and apoptosis of osteoblasts, osteoclasts, and chondrocytes, potentially reducing the rate of disability in people with RA [17]. The role of SIRTs in rheumatoid arthritis is summarized in Table 1.

### 3.1. Role of SIRT1 in RA Pathogenesis

SIRT1 is the best-known member of the SIRT family. It is an enzyme located in the cell nucleus, but it can be transported to the cytosol via two nuclear localization sequences (NLS) and nuclear export signals (NES) [30]. SIRT1 controls inflammation, oxidative stress, immune responses, cell differentiation, and proliferation, as well as cell metabolism, and is therefore likely involved in autoimmune diseases (ADs).

SIRT1 affects many types of cells involved in the development and progression of rheumatoid arthritis, such as chondrocytes, synovial fibroblasts (FLs), macrophages, and T cells (Tc) [31]. SIRT1 is crucial in the regulation of bone metabolism. Chondrocytes are among the cells most frequently damaged in RA, and increased chondrocyte apoptosis has been found both in an animal model of RA [32] and in patients with RA [33]. Macrophages and FLs activate chondrocytes to produce tumor necrosis factor-α (TNF-α) and IL-1β [34], which affect the proliferation and invasion of FLs [35]. One method that slows the progression of RA is based on promoting apoptosis and inhibiting proliferation, invasion, and migration of RA synovial fibroblasts (FLs or RAFLs) cells. SIRT1 activation results in FL apoptosis, mediated by caspase-3 and PI3K/Akt pathway signaling. Overexpression of SIRT1 reduces inducible expression of inflammatory cytokines and osteoblast apoptosis through appropriate NF-κB and p53 signaling—a critical step in cell apoptosis. Deacetylation of the p53 protein by SIRT1 reduces its DNA-binding activity and results in reduced apoptosis. Bax, which is a protein of the Bcl2 family, is located in the cytosol and translocated to the mitochondria after induction of apoptosis; it can be upregulated and transcriptionally activated by the p53 protein [36]. Activation of SIRT1/AMPKα signaling exerts anti-inflammatory effects by regulating M1 and M2 phenotype macrophage polarization, thereby reducing inflammatory responses in RA. SIRT1 signaling should therefore be seen as a therapeutic target in RA. However, no direct evidence has supported the role of SIRT1 in macrophage polarization. Recent findings show that synovial fluid in RA patients contains high levels of M1 macrophage-derived mediators but low levels of M2 macrophage-derived mediators [37]. In vivo studies on heterozygous SIRT1 mice (female) showed a significant decrease in bone density in these mice, which shows that SIRT1 regulates bone metabolism [38]; this is of great importance in the context of RA. Some studies suggest that SIRT1 can inhibit the production of inflammatory cytokines, mainly TNF-α, IL-1β, and IL-6, by influencing the activity of nuclear transcription factor NF-κB, which is involved in inflammatory and immune responses [39,40]. SIRT1 levels in human synovial fibroblasts and chondrocytes obtained from RA patients are higher than in healthy control samples [18,19]. A reduction in SIRT1 expression resulted in a decrease in inflammatory cytokine levels. These results suggest that SIRT1 may be an important regulator of RA inflammation and therapeutically beneficial for RA patients, as it is associated with the inhibition of synovial hypertrophy and inflammation [18]. In contrast, the function of SIRT1 in chondrocytes and synovial fibroblasts during arthritis is unclear and requires further study.

Macrophages and RAFLs are the most important cells in the pathogenesis of RA, invading and degrading adjacent cartilage and bone. Together with RAFLs, they generate inflammation and joint damage and produce pro-inflammatory cytokines and matrix-degrading molecules [41]. SIRT1 is involved in macrophage regulation in inflammation by inhibiting the activity of transcription factors such as activator protein-1 (AP-1) and NF-κB, which are essential for the expression of many inflammatory cytokines. Inhibition of AP-1 activity contributes to the relief of RA symptoms and helps alleviate the disease. Transcription factors, including NF-κB, signal transducer and activator of transcription 3 (STAT3), and interferon regulatory factor 3 (IRF3), which are involved in the production and expression of neutrophil-secreted chemoattractants, have been reported to be substrates for SIRT1 in other cell types. Therefore, it is possible that Sirt1 control the secretion of IL-8 and IFN-γ by neutrophils during RA. Studies by Stein et al. [42] in myeloid-specific Sirt1 gene knockout mice showed that macrophages produce more pro-inflammatory cytokines such as TNF-α, IL-6, and IL-1β in response to infection and inflammation. In addition, SIRT1 has a profound effect on macrophage adhesion and migration during the inflammatory response through its interaction with surface molecules, i.e., intercellular adhesion molecule 1 (ICAM-1), a major regulator of cellular responses in inflammation [42]. Macrophages have been recognized as a key mediator of inflammation in RA, but recent studies indicate that SIRT1 is not only a critical immunosuppressor of macrophage activation but also of T cells.

Extensive research has been carried out to show the role of T cells (especially their activation) in RA pathogenesis [43]. Studies confirm that CD4+ T cells have an important role in the chronic autoimmune response in RA. Interestingly, SIRT1 levels are increased in activated T cells compared to mature naïve T cells, and SIRT1-deficient mice show greater cell proliferation and cytokine production [44]. SIRT1 is described as a negative regulator of T-cell activation, as demonstrated by in vitro and in vivo studies. T cells can recognize antigens, such as collagen, which have been found to be RA initiators. This was confirmed by studies on a group of RA patients in whom collagen-specific T cells were found in peripheral blood samples [45]. SIRT1 inhibition in RA mice model (CIA) decreases the proliferation of Th1 and Th17 cells, as well as dendritic cells (DCs) [46].

**Figure 1:**
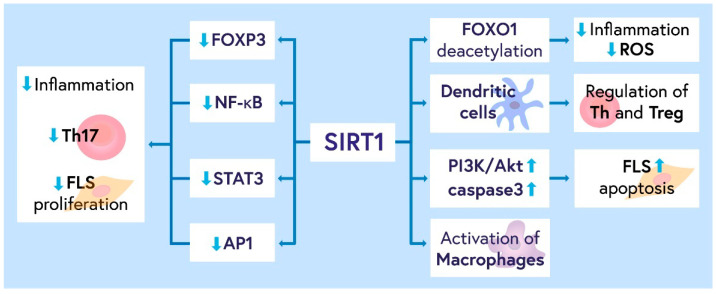
The role of SIRT1 in RA and other autoimmune diseases. SIRT1 could decrease inflammation by inhibiting transcriptional factors such as NF-κB, STAT3, AP-1, FoxP3, FOXO1 deacetylation, and macrophage activity inhibition. By regulating dendritic cells, SIRT1 could control the response mediated by Th and Treg cells. The FLs proliferation and apoptosis are controlled by SIRT1 activity through PI3K/Akt, caspase 3, and NF-κB pathways. Abbreviations: FLs, synoviocytes fibroblasts; AP1, activator protein 1; ROS, reactive oxygen species; Th, T helper cells; Treg, T regulatory cells; Th17, T helper 17 cells; FOX, forkhead box; STAT3, signal transducer and activator of transcription 3; NF-κB, nuclear factor kappa B; PI3K/Akt, phosphatidylinositol 3-kinase (PI3K)/protein kinase B (AKT) signaling pathway; SIRT1, sirtuin 1.

Future research should focus on elucidating the molecular pathways and targets controlled by SIRT1. This may provide a more precise and effective treatment tool for RA, limiting the adverse effects of therapy. The role of SIRT1 in RA, as an autoimmune disease, is schematically shown in Figure 1.

### 3.2. Role of SIRT2-SIRT 7 in RA Pathogenesis

The roles of SIRT2-7 in the pathogenesis of RA have been very poorly studied, and research results are often inconsistent.

Sirtuin 2 (SIRT2), located in the cell nucleus and cytosol, and SIRT3, located in the mitochondrial matrix, play an important role in regulating mitochondrial metabolism, including amino acid metabolism, fatty acid oxidation, the tricarboxylic acid cycle, and the urea cycle [20]. SIRT2 and SIRT3 have been detected in tissues such as the kidney, heart, brain, and liver [47]. These SIRTs have been shown to play a role in aging, neurodegenerative diseases, and the process of apoptosis. To date, the involvement of these SIRTs in RA has not been elucidated, and the results of studies to date are often contradictory. It was shown that mRNA expression of SIRT3 was higher in RA patients than in healthy individuals, while mRNA expression of SIRT2 was lower in the RA group than in healthy subjects [48,49]. In addition, mRNA expression of SIRT2 and SIRT3 positively correlated with RA activity. On the other hand, other authors observed decreased levels of mitochondrial expression of SIRT3 in RA patients [12]. The expression of SIRT2 and SIRT3 was significantly associated with positive anti-CCP (cyclic citrullinated peptide) status, increased ESR (erythrocyte sedimentation rate), and increased CRP (C-reactive protein) levels [12,49]. Dysregulation of SIRT2 exacerbates the severity of collagen-induced arthritis (CIA). Studies indicate that SIRT2 deficiency facilitates the development of collagen-induced arthritis by increasing the production of pro-inflammatory cytokines such as IL-1β, IL-6, and TNF-α [50]. Decreased levels of SIRT3 result in decreased levels of mitochondrial SOD2, increased oxidative stress, and ultimately lead to increased osteoarthritis [12].

Sirtuin 4 (SIRT4), located in the mitochondria, is an enzymatic protein that exhibits NAD+-dependent deacetylase activity—an important function in human inflammatory diseases. One of its key functions is to inhibit the activity of mitochondrial glutamate dehydrogenase 1 (GLUD1) by ADP ribosylation activity while reducing insulin secretion in response to amino acids [51]. Changes in insulin levels by SIRT4 may also be the cause of an interaction with an insulin-degrading enzyme (IDE). Whether this is via direct interaction or via ADP ribosylation has not been established. It is worth emphasizing that SIRT4 does not show deacetylase activity [52]. In contrast, deacetylation of malonyl-CoA decarboxylase by SIRT4 inhibits fatty acid oxidation in muscle and liver cells. SIRT4 also represses the transcription of genes underlying β-oxidation, such as peroxisome proliferator-activated receptor alpha (PPAR-α) [53]. SIRT4 overexpression has been shown to increase TNF-α and IL-6 secretion and accelerate bone destruction in patients with osteoarthritis [51,54]. Studies were also performed on the effects of SIRT4 on human cartilage cells from osteoarthritis patients. Chondrocytes treated with SIRT4 showed a significant increase in collagen II and aggrecan. Inhibition of reactive oxygen species (ROS) was also observed, thereby reducing the inflammatory response [51]. These studies indicate that SIRT4 is involved in the development of RA, and its overexpression contributes to decreasing the inflammatory response and oxidative stress.

Sirtuin 5 (SIRT5) regulates cellular metabolism by participating in the β-oxidation of fatty acids, the tricarboxylic acid (TCA) cycle, and glycolysis [25]. SIRT5 affects macrophages, inhibiting inflammation by activating PKM2 kinase activity and blocking IL-1β production in macrophages [55]. It has been shown that SIRT5 deficiency increases disease severity in rats with adjuvant-induced arthritis [55]. In addition, SIRT5 can deacetylate the mitochondrial intermembrane space (IMS) cytochrome c protein in vitro, which is involved in oxidative metabolism and apoptosis [56]. Studies have shown that SIRT5 mRNA expression is significantly reduced in RA patients compared to healthy subjects [24]. In addition, SIRT5 has been shown to regulate chondrocyte metabolism, including amino acid metabolism, the tricarboxylic amide cycle, and glycolysis [57]. Studies have shown that SIRT5 mRNA is significantly reduced in RA patients compared to healthy individuals. It was also shown that blocking SIRT5 significantly enhances the release of TNF-α and IL-1β, which are involved in the development of RA. SIRT5 deficiency increased the severity of this disease [24].

**Figure 2:**
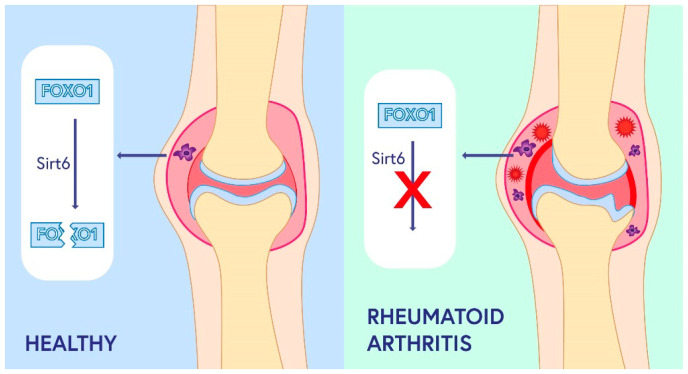
The role of sirtuin 6 in rheumatoid arthritis. In healthy joints, SIRT6 deacetylates FOXO1, leading to its transport into the cytoplasm and subsequent degradation. In RA patients, deacetylation of FOXO1 (Forkhead box protein O1) by SIRT6 in macrophages is impaired, resulting in the activation of immune cells, increased inflammation, and tissue destruction.

Sirtuin 6 influences a number of processes that may be involved in the development of RA. SIRT6 is involved in fatty acid metabolism and influences the secretion of TNF-α, a cytokine that plays a key role in RA pathogenesis; moreover, SIRT6 modulates NF-κB-related metabolic pathways [58]. A number of pro-inflammatory mediators regulated by NF-κB are involved in the pathogenesis of RA. SIRT6 exhibits anti-inflammatory effects by inhibiting NF-κB-dependent pathways. This was confirmed in mice with induced arthritis, in which administration of SIRT6 resulted in reduced inflammation and decreased secretion of pro-inflammatory cytokines [26]. Moreover, it was shown that SIRT6 inhibited osteoclast differentiation induced by GM-CSF (granulocyte-macrophage colony-stimulating factor) and RANKL (receptor activator of nuclear factor kappa-B ligand) [59]. These results suggest that blocking the NF-κB pathways by SIRT6 in rheumatoid arthritis reduces both the inflammatory response and leads to a reduction in joint destruction. Moreover, myeloid SIRT6 deficiency has been shown to affect the development and exacerbation of RA in mice by increasing the migratory potential of macrophages [27]. In healthy joints, SIRT6 deacetylates FOXO1, leading to its transport to cytoplasm and degradation [60]. In RA patients, deacetylation of FOXO1 by SIRT6 in macrophages is impaired, resulting in the activation of immune cells, increased inflammation, and tissue destruction [27] (Figure 2). Zhang et al. showed that SIRT6 in RA-fibroblast-like synoviocytes suppresses cell proliferation and inflammation and induces apoptosis, thereby alleviating RA symptoms [61].

SIRT7 is the most recently discovered and least studied SIRT [62], mainly involved in the deacetylation of lysine residues at the K18 position of histone H3 (H3K18Ac) [63]. Like other SIRTs, it is involved in the regulation of many metabolic processes. High expression of SIRT7 has been found in metabolically active tissues such as the liver, testes, and spleen [64]. To date, the role of this SIRT in the pathogenesis of RA has not been investigated. However, SIRT7 has been shown to affect chondrocyte metabolism, protecting chondroid tissue from degeneration [65]. SIRT7 positively regulates rDNA transcription and can regulate both RNA Pol II and RNA Pol I transcription, acting with various other factors [27].

## 4. Regulation of SIRT1 as a Potential Target in RA Therapy

The modulation of SIRT proteins, particularly SIRT1 activity, appears to be important in the pathogenesis of rheumatoid arthritis. Below, we describe the most well-known SIRT activating factors and actions that contribute to reducing RA symptoms and improving patient quality of life and well-being, making SIRT a potential target for RA therapy.

### 4.1. Resveratrol in RA Therapy

Resveratrol (RSV) is a natural phytoalexin polyphenol that affects cell sensitivity to insulin and improves blood vessel function. It is also the best-known and most-studied activator of the SIRT enzyme. For years, resveratrol has been considered a potential antioxidant drug for the treatment of various autoimmune diseases, as well as in anti-cancer therapy. Many of these effects are due to the modulation of SIRT1 targets, such as peroxisome proliferator-activated receptor co-activator-1α (PGC1α) and NF-κB. Studies have shown that resveratrol can also activate AMP-activated protein kinase (AMPK), inhibit cyclooxygenases, and affect many other enzymes [66].

**Figure 3:**
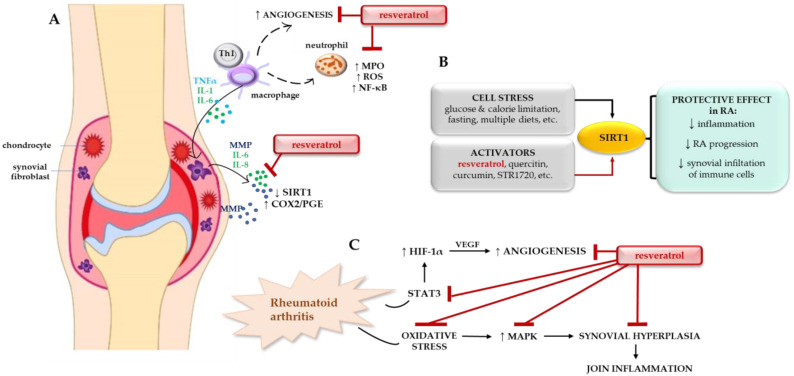
Prevention role of resveratrol in rheumatoid arthritis. (**A**) The pathophysiological mechanism of rheumatoid arthritis (RA) controlled by environmental factors, gene interactions, and the immune cells response, (**B**) SIRT1 activating factors and its protective effects in RA, (**C**) Resveratrol’s role in counteracting RA development. Abbreviations: COX2, cyclooxygenase 2; IL, interleukin; TNFα, tumor necrosis factor alfa; MMP, metalloproteinases; MPO, myeloperoxidase; NF-κB, nuclear factor kappa B; PGE prostaglandin E; ROS, reactive oxygen species; VEGF, vascular endothelial growth factor; SIRT1, sirtuin 1; MAPK, mitogen-activated protein kinases; HIF-1α, hypoxia-inducible factor 1-alpha; STAT3, signal transducer and activator of transcription 3; Th1, T helper cells.

Resveratrol-induced activation of SIRT1 has been shown to have considerable therapeutic potential in the treatment of patients with autoimmune inflammatory diseases, including RA [47]. In studies conducted by Yang et al., it was observed that resveratrol-treated RSC-364 cells (lipopolysaccharides-induced fibroblast-like synoviocytes) showed both G0/G1 cell cycle arrest and increased levels of apoptosis. These results confirmed the preventive role of resveratrol in the progression of RA. The mechanism of action of resveratrol consists of blocking MAPK signaling pathways, which can inhibit the inflammatory response and cell proliferation, and, moreover, activate apoptosis in synovial tissue, with a simultaneous reduction in angiogenesis with the participation of hypoxia-inducible factor 1 alpha (HIF-1α) [47]. As such, resveratrol appears to have excellent potential in RA therapy. This was confirmed by the results of in vivo studies showing that RSV can reduce bone loss and promote bone formation (osteogenesis) by enhancing resistance to oxidative stress in a mouse model [67]. The prevention role of resveratrol in rheumatoid arthritis is schematically shown in Figure 3.

RSV can promote osteogenesis by activating the SIRT1/FOXO1 signaling pathway, which is a recently described target for the prevention and treatment of osteoporosis. In this experimental model, SIRT1 was upregulated and the level of Nu-FOXO1, which had high redox-regulated transcriptional activity, was also significantly increased. The beneficial effect of RSV has been demonstrated in a rat RA model [68], as RSV supplementation reduced serum antioxidant enzyme levels, arthritic status, paw swelling, synovial hyperplasia, inflammatory cell infiltration, and cartilage degradation in these animals. RSV also inhibited the proliferation of synoviocytes in synovial tissues. Further studies have shown that RSV acts through the activation of the silent information regulator 1 (SIRT1)/nuclear erythroid factor-related factor 2 (Nrf2) signaling pathway. RSV also affects the immune system, which is also involved in the progression of RA disease.

Resveratrol contributes to the modulation of innate and adaptive immunity by stimulating the activation of macrophages, NK cells, and T cells, as well as the inhibitory regulation of CD4+ CD25+ T lymphocytes [69]. Macrophages, along with RAFL cells, degrade cartilage and bone and are a key factor responsible for the development and pathogenesis of RA. Studies have shown that SIRT1, by affecting cell cycle and longevity pathways, is a key regulator of macrophage self-renewal and may be an important parameter of aging [70]. Resveratrol can regulate the expression of TLR4, which plays a fundamental role in pathogen recognition and activation of innate immunity. Therefore, RSV may affect TLR-mediated inflammatory responses in rheumatoid arthritis [71]. Activation of SIRT1 by RSV inhibits the acetylation of p65/RelA (a member of the NF-κB family), which regulates leukocyte activation and controls inflammatory cytokine signaling. This activation reduces the expression of inflammatory factors, such as TNF-α, interleukins (IL-1 and IL-6), metalloproteinases (MMP1 and MMP3), and cyclooxygenase (COX-2) [72].

Health benefits through SIRT1 activation are provided not only by resveratrol, as a naturally derived product, but also by other plant flavanols from the polyphenolic flavonoid group, such as quercetin, apigenin, catechin, epicatechin, theobromine, curcumin, soy isoflavones, sulforaphane, olivetol, isothiocyanates, piceatannol, cinnamon, and fisetin [73]. These are the most popular and most studied flavonoid derivatives that have emerged in the context of SIRT modulation. In fact, small molecules appear to be the most effective modulators of SIRT, especially for SIRT1, but also for SIRT3 and SIRT6 [74,75]. Based on this information, molecules that stimulate sirtuin activities more strongly than resveratrol have also been developed, e.g., SRT1720, SRT2104, and SRT2379. However, their structure is not related to resveratrol. This shows how much demand there is for drugs and/or supplements that can help patients, perhaps including RA patients.

As a matter of fact, RA treatment is based on conventional and biological therapies, including TNF inhibitors (adalimumab, certolizumab pegol, etanercept, golimumab, and infliximab), abatacept, rituximab, IL-6 inhibitors (tocilizumab and sarilumab), biosimilars, and small oral molecules (the JAK inhibitors tofacitinib and baricitinib) [76,77]. The goal of currently approved therapies in the treatment of RA is the use of biological disease-modifying antirheumatic drugs (bDMARDs), represent a breakthrough in RA treatment, and targeted synthetic DMARDs—based on JAKs/MAPKs/NF-κB/SYK-BTK signaling inhibitors [78].

### 4.2. Healthy Diet

SIRT1 expression can be altered by intracellular and environmental factors, including natural and synthetic SIRT1 agonists (like resveratrol, SRT1720), inhibitors (tenovin, sirtinol), as well as cellular stress caused by starvation and glucose or caloric restriction. Therefore, one of the factors that can regulate and affect the activity of sirtuins—as proteins responsible for cellular metabolism and sensitive to cellular stress—is a diet based on nutrient and energy restriction.

Pentosidine is one of the systemic oxidative stress indicators [79]. It has been reported that the level of pentosidine (in serum, urine, and synovial fluid) is correlated with the activities of RA [80]. Urinary pentosidine (glycation end product) is a glycoxidation damage biomarker, and the glycoxidation reaction of protein is thought to contribute to the cross-linking of tissue proteins, gumming up tissues, making them stiffer and less elastic, and so causing connective tissues to become leathery. Formation of pentosidine is accelerated in the increased sugar concentrations, which is why dietary regimens such as fasting and caloric restriction, or even a vegetarian diet, have beneficial effects by improving symptoms of patients with RA. Recently, it has been reported that dietary regimens such as fasting/caloric restriction diet [80,81,82,83,84,85], vegetarian and gluten-free vegan diet [85,86,87,88,89], and Mediterranean sea diet [90,91], have suppressive and beneficial effects, improving the condition and well-being of patients with SIRT-dependent diseases, such as RA. Studies have shown that RA activity decreased with a low-energy diet (lasting 54 days), accompanied by a decrease in urinary pentosidine levels—associated with rheumatoid arthritis activity [80]. There are results of two studies conducted by a group of German researchers [81,83], in which RA patients were subjected to a period of 8 days fasting, with a daily limit of 300 kcal). They showed a slight reduction in the RA disease activity scores compared to the control group. A clinical trial evaluating the effectiveness of therapeutic fasting with time-limited eating hours in patients with RA is currently underway (NCT03856190). Fasting as one of the dietary modifications has been actively investigated as an alternative therapy in RA. Several clinical trials have been conducted to test its efficacy [84,85]. Patients in the fasting group showed significant improvements in all clinical parameters and some laboratory parameters, i.e., red blood cell sedimentation rate (ESR) and C-reactive protein (CRP), which are correlated with RA disease severity [86]. Vegetarian diets eliminate meat products, while vegan diets are void of all animal-based food products (eggs, dairy, and seafood). These diets gained popularity in RA patients as a potential therapeutic tool and demonstrated positive results in clinical trials. McDougall et al. [88] found that a low-fat vegan diet significantly decreased patients’ pain scores, the number of tender, swollen joints, and CRP levels. A 6-week study with RA patients (130 female) on the Mediterranean sea diet (MD)-typically high in vegetables, fruits, whole grains, nuts, and monounsaturated fats, such as olive oil, and low in red meat, demonstrated significant improvement in RA symptom outcomes and disease activity scores, but with no change in CRP, compared to controls [90]. Johansson et al. [91] found that higher adherence to an MD was associated with a lower odds ratio of developing seropositive RA (interestingly, only among the male group). The ketogenic diet (KD) involves restricting carbohydrates (to a maximum of 5–10% of total daily caloric intake), which shifts metabolism toward ketone bodies. Research and literature on KD and its anti-inflammatory properties in rheumatic diseases are still limited but have shown promise in helping patients lose weight, reduce insulin requirements in diabetes, and control the metabolic disease that is common in RA patients [92].

It is important to note that calorie-restricted diets improve the health of patients with RA and reduce disease symptoms (it can be said that prevents RA), but so far, there are no data showing clearly that diet or caloric restriction changes the expression of SITRs in RA patients’ tissues. All data and observations in patients with other age-related diseases suggest that perhaps the calorie restriction or diets also works on sirtuins in RA; however, it requires further research. So far, the data are incomplete.

## 5. Conclusions

SIRTs influence a number of processes that may be involved in the development of RA. To date, the role of all SIRTs in RA pathogenesis is not well-understood, and the mechanisms by which SIRTs may affect RA development have not been fully elucidated. SIRTs can affect immune cells, such as T and B lymphocytes, macrophages (regulators of macrophage self-renewal), neutrophils, and chondrocytes, which are all directly involved in the development of inflammation in RA. They also regulate pathways related to NF-κB and STAT3, which are associated with the synthesis of pro-inflammatory mediators in RA. The mechanism of SIRT action is based on the deacetylation of histones and many transcription factors, including p65, p53, STAT3, the FOXO family, PGC1α, leading to the repression of transcription. SIRTs can promote cell survival by impeding apoptosis and cell death in response to DNA damage and oxidative stress. Previous studies have suggested the involvement of SIRTs in the immune response, apoptosis, and many other processes that induce RA. Research has shown that by activating SIRT (e.g., SIRT1), we can limit the development of autoimmune diseases such as RA. It is now believed that small molecules can have a positive therapeutic effect to support the treatment of patients with RA by activating information SIRTs and their pathways. However, understanding the specific role of SIRTs in the development of RA requires further research.

## Figures and Tables

**Table 1:** The role of sirtuins in rheumatoid arthritis.

Name	Function	References
SIRT1	- inhibition of cell proliferation and migration; - enhancement of caspase-3 and -8 activity; - decreased secretion of TNF-α, IL-6, IL-8, and IL-1β; - inhibition of the NF-κB pathway;	[18,19]
SIRT2	- regulation of mitochondrial metabolism; - amino acid metabolism, fatty acid oxidation; - the tricarboxylic acid cycle;	[20]
SIRT3	- regulation of mitochondrial metabolism; - amino acid metabolism, fatty acid oxidation; - the tricarboxylic acid cycle;	[21]
SIRT4	- control of mitochondrial function and cell cycle checkpoints; - DLAT regulation; - enzymatic activity correlated with anti-CCP, ESR, and CRP;	[22,23]
SIRT5	- increasing the secretion of TNF-α and IL-6 cytokines, - accelerating the bone destruction process; - activation of PKM2 kinase and blocking of IL-1β production in macrophages;	[24,25]
SIRT6	- deacetylation of histones H3K9 and H3K56; - regulation of protein secretion and movement across the cell membrane; - attenuation of NF-κB transactivation;	[26,27]
SIRT7	- deacetylation of the lysine at position K18 in histone H3; - stabilization of cancer cells’ transformation state; - regulation of RNA Pol II and RNA Pol I transcription;	[28,29]

## Data Availability

Not applicable.
